# Pancreatitis as a Rare Complication of Dengue Fever: A Case Report and Review of the Literature

**DOI:** 10.7759/cureus.82725

**Published:** 2025-04-21

**Authors:** Maitha K Alnuaimi, Jelal A Alsubai, Abdulaziz Alnuaimi

**Affiliations:** 1 Internal Medicine, Tawam Hospital, Abu Dhabi, ARE; 2 Gastroenterology, Tawam Hospital, Abu Dhabi, ARE

**Keywords:** acute pancreatitis, case report, dengue fever, rare complication, tropical disease

## Abstract

Dengue fever is a prevalent mosquito-borne viral infection in tropical and subtropical regions. High-grade fever, myalgia, arthralgia, and hemorrhagic manifestations are among its classic clinical features. Acute pancreatitis, however, remains an exceptionally rare complication. Awareness of this possibility is crucial, as early recognition and appropriate management can significantly improve patient outcomes. A previously healthy 44-year-old woman presented with a five-day history of severe epigastric pain radiating to the back, high-grade fever (39°C), profuse sweating, and persistent greenish vomiting. Laboratory results revealed leukopenia, elevated liver enzymes, and markedly raised pancreatic enzymes (amylase and lipase). Dengue infection was confirmed by polymerase chain reaction. Imaging demonstrated minimal biliary sludge and mild pancreatic edema without evidence of gallstones or necrosis. She was managed supportively with intravenous fluids, analgesics, and antiemetics, and her condition improved rapidly. By the fourth day of hospitalization, she was discharged in stable condition with dietary advice. Acute pancreatitis complicating dengue is rarely reported, with proposed mechanisms involving direct viral invasion, immune-mediated damage, or ischemic injury from increased vascular permeability. This case underscores the need to consider pancreatitis in patients with dengue who experience severe or persistent abdominal pain. A high index of suspicion is paramount to ensure early diagnosis, prompt supportive treatment, and favorable patient outcomes. This report highlights acute pancreatitis as a rare but important complication of dengue fever. Clinicians practicing in dengue-endemic areas should maintain vigilance for this diagnosis to facilitate early intervention. Strengthening physician awareness may influence guidelines and promote more timely identification of this unusual yet significant complication.

## Introduction

Dengue fever is a mosquito-borne illness caused by the dengue virus, transmitted predominantly by the Aedes aegypti mosquito. Globally, dengue is a major public health concern; the World Health Organization (WHO) estimates that around 390 million dengue infections occur each year, primarily in tropical and subtropical regions [1]. Clinically, dengue typically presents with an acute onset of high-grade fever, severe headache, retro-orbital pain, rash, and musculoskeletal pain [2]. Severe disease can manifest as dengue hemorrhagic fever or dengue shock syndrome, but atypical presentations affecting hepatic, neurological, or cardiac systems are also recognized [3]. Acute pancreatitis is among these less common complications, and its incidence in dengue is not well-defined, though it appears to be very low based on scarce reports [4,5].

Proposed pathophysiological mechanisms for dengue-related pancreatitis include direct viral invasion, immune-mediated inflammatory damage, and microvascular ischemia due to plasma leakage [6]. Overlapping gastrointestinal symptoms, such as nausea, vomiting, and abdominal pain in dengue, can mask or delay the diagnosis of pancreatitis unless pancreatic enzymes are specifically assessed. Here, we present a case of acute pancreatitis in a patient with confirmed dengue, highlighting the importance of maintaining a high index of suspicion for this rare complication.

## Case presentation

A 44-year-old woman, with no significant past medical history, presented with a five-day history of severe epigastric pain radiating to her back. The pain was constant, unrelated to meals, and unrelieved by over-the-counter analgesics, including paracetamol and tramadol. She described intermittent high-grade fever (peaking at 39°C), profuse sweating, and persistent greenish vomiting. No changes in bowel habits or urinary symptoms were reported. She had neither a history of alcohol use nor of previous similar episodes.

Her initial management for a presumed dengue infection at a local hospital involved supportive care, but she was referred to our tertiary center due to persistent symptoms. On examination, she was hemodynamically stable, with a temperature of 36.6°C, heart rate of 75 beats per minute, blood pressure of 108/65 mercury (mmHg), respiratory rate of 18 breaths per minute, and oxygen saturation of 98% on room air. She appeared alert, though in mild discomfort from epigastric tenderness. No organomegaly or palpable masses were noted.

Laboratory investigations showed leukopenia (white blood cell (WBC) of 3.9 × 10^9^/L), elevated transaminases (alanine transaminase (ALT) of 202 IU/L and serum aspartate aminotransferase (AST) of 158 IU/L), and mildly raised bilirubin levels (total bilirubin of 5.8 µmol/L, direct bilirubin of 2.8 µmol/L). Serum pancreatic enzymes were significantly increased (amylase of 281 units/L and lipase of 357 IU/L), raising the suspicion of acute pancreatitis. Dengue virus infection was confirmed by polymerase chain reaction (PCR). Table 1 provides a summary of the key laboratory findings, including hematological and biochemical parameters, observed during the patient's initial evaluation.

**Table 1:** Summary of key laboratory findings

Laboratory Test	Result	Reference Range
White blood cell (WBC) count	3.9 × 10^9^/L	4.0–11.0 × 10^9^/L
Hemoglobin (Hb)	129 g/L	120–160 g/L (female)
Platelets	180 × 10^9^/L	150–400 × 10^9^/L
Alanine transaminase (ALT)	202 IU/L	7–56 IU/L
Aspartate transaminase (AST)	158 IU/L	10–40 IU/L
Alkaline phosphatase (ALP)	80 IU/L	44–147 IU/L
Total bilirubin	5.8 µmol/L	0.0–17.1 µmol/L
Direct bilirubin	2.8 µmol/L	0.0–4.3 µmol/L
Serum amylase	281 units/L	23–85 units/L
Serum lipase	357 IU/L	0–160 IU/L
C-reactive protein (CRP)	<10 mg/L	<10 mg/L
Triglycerides	1.57 mmol/L	<1.7 mmol/L
Low-density lipoprotein (LDL) cholesterol	0.84 mmol/L	<3.5 mmol/L
High-density lipoprotein (HDL) cholesterol	1.00 mmol/L	>1.0 mmol/L
Total cholesterol	2.56 mmol/L	<5.2 mmol/L

**Figure 1:**
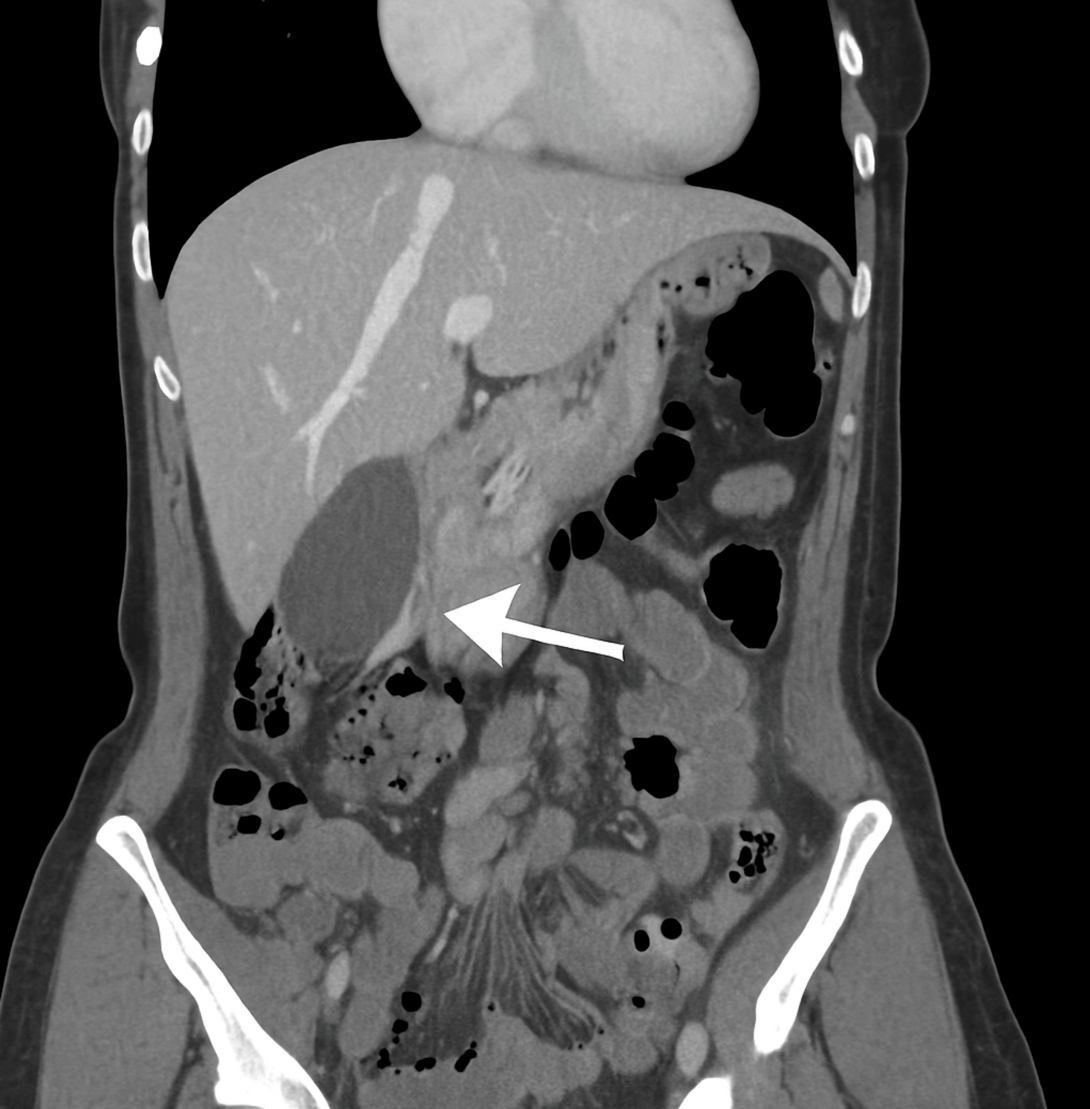
Coronal CT scan showing mild pancreatic edema This coronal view highlights the edematous pancreas (white arrow) without evidence of necrosis or pseudocyst formation.

**Figure 2:**
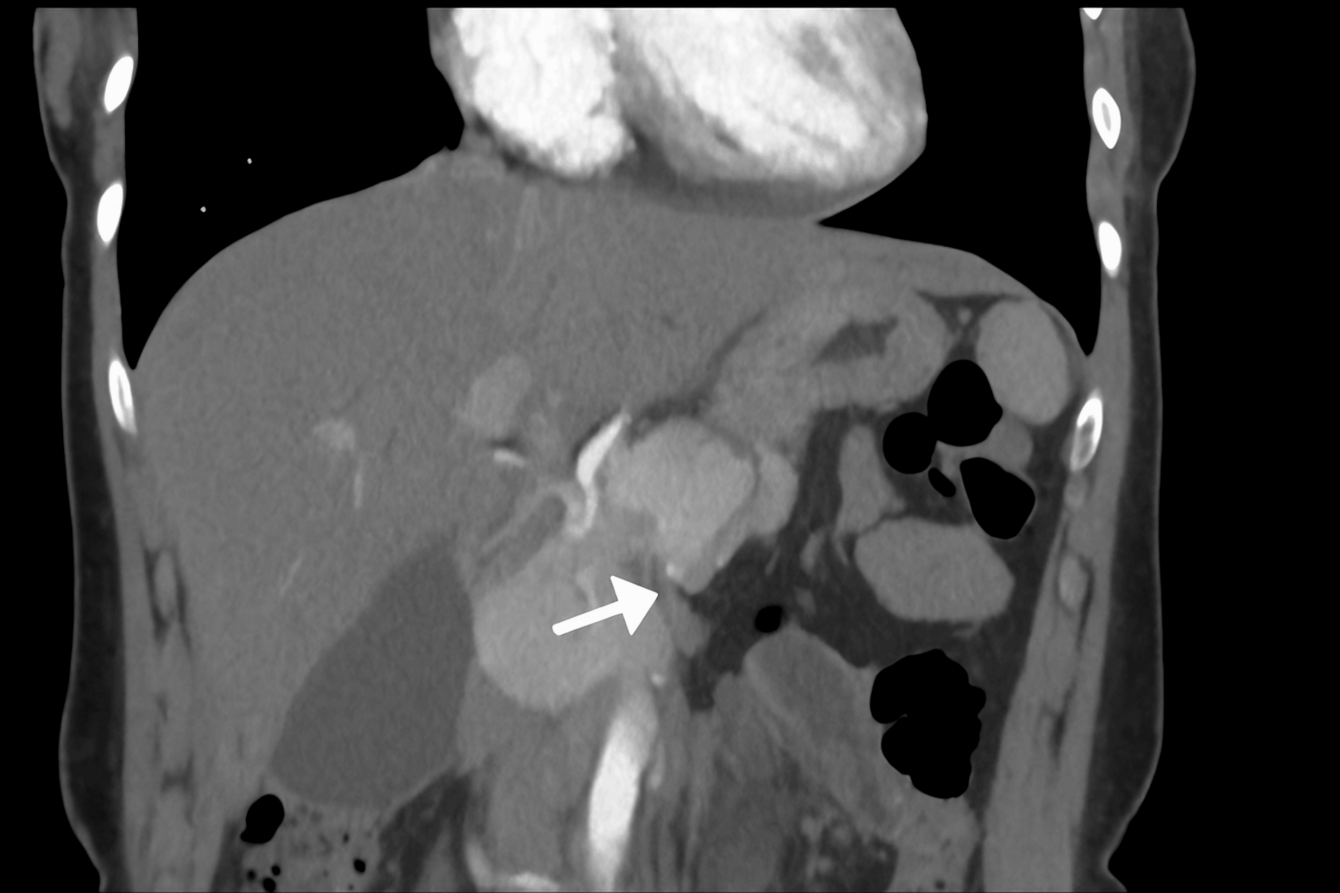
Coronal CT scan demonstrating fluid around the pancreas Another coronal slice reveals mild peripancreatic fluid (white arrow). The surrounding structures appear unremarkable.

**Figure 3:**
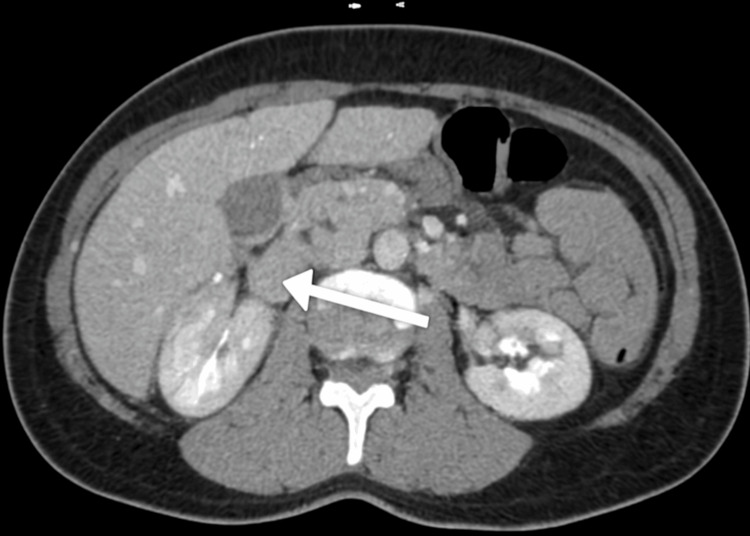
Axial CT scan of the abdomen revealing pancreatic edema An axial slice depicting the swollen pancreatic parenchyma (white arrow) with no signs of gallstones or biliary dilation.

Abdominal ultrasound revealed minimal biliary sludge but no gallstones or biliary dilation. A contrast-enhanced computed tomography (CT) scan of the abdomen showed mild pancreatic edema and minimal free fluid in the pelvis, with no necrosis or pseudocyst formation. A simple right ovarian cyst measuring up to 3 cm was also noted (Figures 1-3).

**Figure 4:**
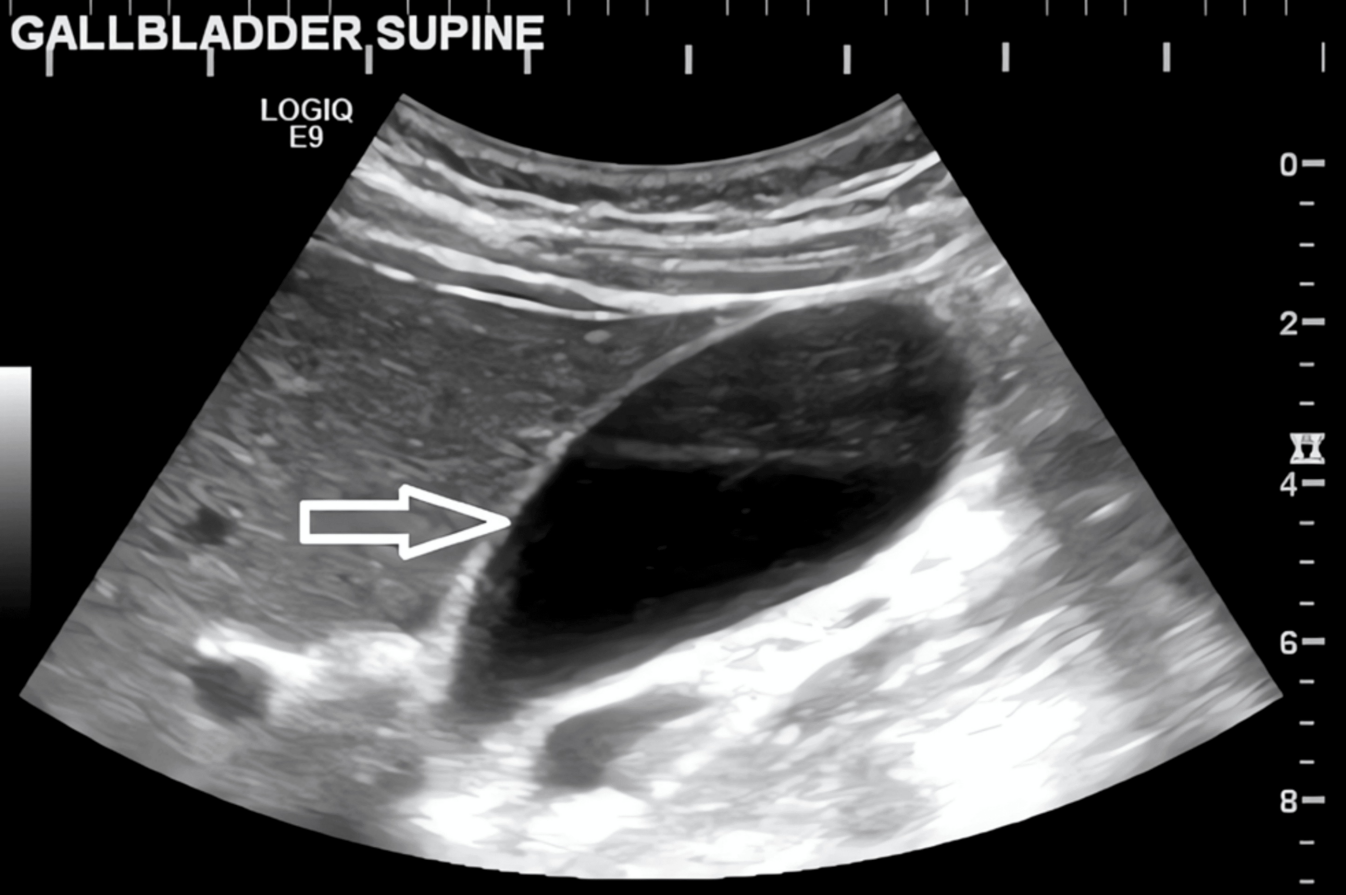
Ultrasound image of the gallbladder showing minimal biliary sludge This ultrasound scan (longitudinal view) demonstrates mildly echogenic material (white arrow) within the gallbladder lumen, consistent with biliary sludge.

**Figure 5:**
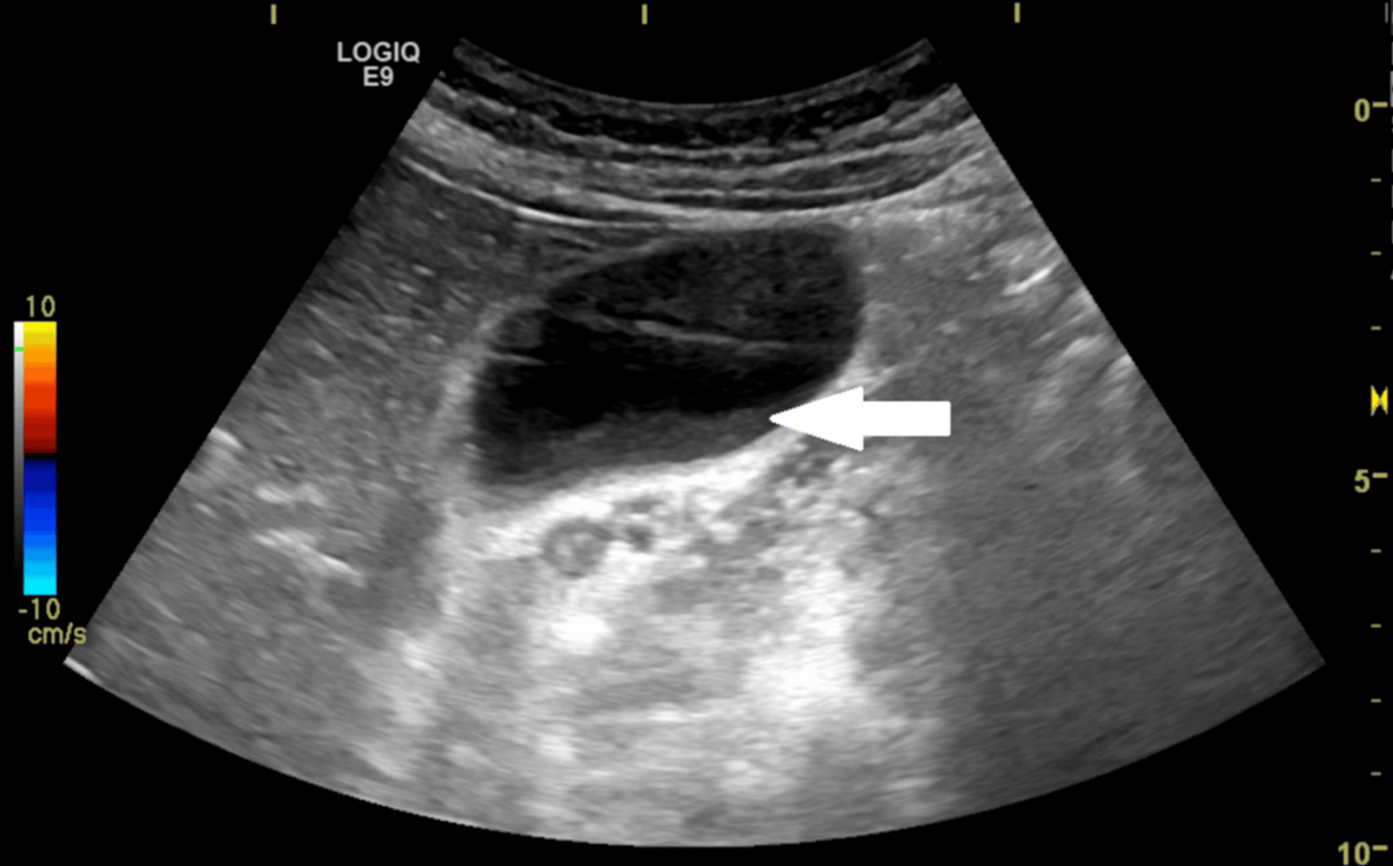
Alternate ultrasound angle confirming the absence of cholelithiasis An additional ultrasound view of the gallbladder (white arrow), further demonstrating the absence of gallstones or biliary tract dilation.

Further ultrasound imaging confirmed minimal biliary sludge but no calculi (Figures 4-5).

Based on the clinical presentation, imaging findings, and significant elevations in pancreatic enzymes (meeting the revised Atlanta classification criteria [7]), the diagnosis of acute pancreatitis secondary to dengue fever was made. Other common etiologies of pancreatitis, such as gallstones, hypertriglyceridemia, and alcohol use, were effectively ruled out.

The patient was managed conservatively with intravenous (IV) fluids for rehydration, analgesics for pain relief, and antiemetics for nausea and vomiting. Over four days, her abdominal pain subsided, vomiting ceased, and she tolerated oral intake. She was discharged in stable condition with advice to continue a low-fat diet. At outpatient follow-up, she remained asymptomatic and had normalized pancreatic enzyme levels.

## Discussion

Acute pancreatitis is an uncommon but notable complication of dengue fever, often overshadowed by more characteristic manifestations, such as high-grade fever, hemorrhagic tendencies, and shock. In this case, the development of epigastric pain radiating to the back, coupled with markedly elevated serum amylase and lipase, was pivotal in diagnosing pancreatitis. Dengue was implicated as the etiology after excluding other common causes of pancreatitis.

While the exact pathophysiology of dengue-associated pancreatitis is not fully elucidated, it may involve direct viral invasion of pancreatic acinar cells, immune-mediated inflammatory damage, or microvascular ischemia due to increased vascular permeability [6]. Abdominal pain is not uncommon in dengue itself, and without specific evaluation of pancreatic enzymes and imaging, pancreatitis can be easily missed.

Most published cases of dengue-related pancreatitis follow a relatively mild clinical course, responding to conservative management with adequate fluid resuscitation, analgesia, and supportive measures [4,5]. In one such instance, an 18-year-old woman from Peru presented during a dengue outbreak with abdominal pain, fever, and mild pancreatitis, drawing attention to the possibility that dengue infection may trigger underdiagnosed pancreatic inflammation [4]. Similarly, a case from Nepal reported a 24-year-old female developing mild acute pancreatitis in the background of dengue fever, highlighting that gallstones and alcohol use were excluded as causes, and suggesting the importance of serological testing for dengue in patients with acute abdominal pain [5]. Of note, a systematic review cited in that report studied 9,365 dengue patients, finding 16% with acute abdomen and 7.7% of those attributed to pancreatitis, which may reflect an underestimated burden in endemic areas. Although most cases remain mild, severe forms have also been documented; for example, a 15-year-old male child developed necrotizing pancreatitis as the first presentation of dengue, requiring aggressive supportive measures and eventually recovering with conservative treatment [8]. Another small series detailed two patients who presented with fever, generalized body aches, and acute abdominal pain, both progressing to pancreatitis confirmed by imaging [9].

Nevertheless, severe cases may occur, warranting careful monitoring for complications such as necrosis, pseudocyst formation, or systemic inflammatory response [10]. Interestingly, a South Indian study reported that 5.8% of dengue patients developed acute pancreatitis, with an overall mortality rate of 1.7% that rose to 7.31% in those with pancreatitis, underscoring increased risk, particularly in individuals aged 51 years and above [11]. However, given the high prevalence of dengue in endemic areas, even a small proportion of patients can represent a significant absolute number of cases. This underscores the importance of considering pancreatitis in patients with persistent or severe abdominal pain during dengue infection, particularly in environments where dengue incidence is high.

Late or missed diagnoses are possible if abdominal symptoms are attributed solely to dengue’s usual clinical picture. The availability of serum lipase/amylase testing and imaging can facilitate prompt recognition. However, resource-limited settings - where dengue is most prevalent - may face constraints in obtaining these diagnostic tools. Further research and epidemiological data are necessary to better clarify risk factors, outcomes, and optimal management strategies for dengue-related pancreatitis.

## Conclusions

Acute pancreatitis is a rare but important complication of dengue fever. This case highlights the significance of thorough evaluation in patients with dengue who develop sustained or intense abdominal pain. Prompt measurement of pancreatic enzymes and judicious imaging studies can expedite recognition and facilitate timely treatment, contributing to improved patient outcomes. Clinicians working in dengue-endemic areas should remain vigilant for this complication. Wider dissemination of such case findings may help shape local guidelines and advocate for enhanced awareness, ultimately improving the standard of care for dengue patients.
